# Alternatives to antibiotics for treatment of mastitis in dairy cows

**DOI:** 10.3389/fvets.2023.1160350

**Published:** 2023-06-19

**Authors:** Xiaoping Li, Chuang Xu, Bingchun Liang, John P. Kastelic, Bo Han, Xiaofang Tong, Jian Gao

**Affiliations:** ^1^Department of Clinical Veterinary Medicine, College of Veterinary Medicine, China Agricultural University, Beijing, China; ^2^Faculty of Veterinary Medicine, University of Calgary, Calgary, AB, Canada

**Keywords:** dairy cows, bovine mastitis, NSAIDs, herbal medicines, antimicrobial peptides, bacteriophages, vaccination

## Abstract

Mastitis is considered the costliest disease on dairy farms and also adversely affects animal welfare. As treatment (and to a lesser extent prevention) of mastitis rely heavily on antibiotics, there are increasing concerns in veterinary and human medicine regarding development of antimicrobial resistance. Furthermore, with genes conferring resistance being capable of transfer to heterologous strains, reducing resistance in strains of animal origin should have positive impacts on humans. This article briefly reviews potential roles of non-steroidal anti-inflammatory drugs (NSAIDs), herbal medicines, antimicrobial peptides (AMPs), bacteriophages and their lytic enzymes, vaccination and other emerging therapies for prevention and treatment of mastitis in dairy cows. Although many of these approaches currently lack proven therapeutic efficacy, at least some may gradually replace antibiotics, especially as drug-resistant bacteria are proliferating globally.

## 1. Introduction

Milk and its derivatives are rich in nutrients and a common food for people of all ages (1). In addition to its nutrient content, milk of BCoV-vaccinated cows had BCoV antibodies and drinking this milk helped people acquire SARS-CoV-2 heterologous antibodies and thus develop passive immunity against COVID-19 (2). Milk antibodies also conferred protection against *rotavirus*, *Shigella flexneri*, *Escherichia coli*, *Clostridium difficile*, *Streptococcus mutans*, *Cryptosporidium parvum*, and *Helicobacter pylori* (3, 4).

**Figure 1:**
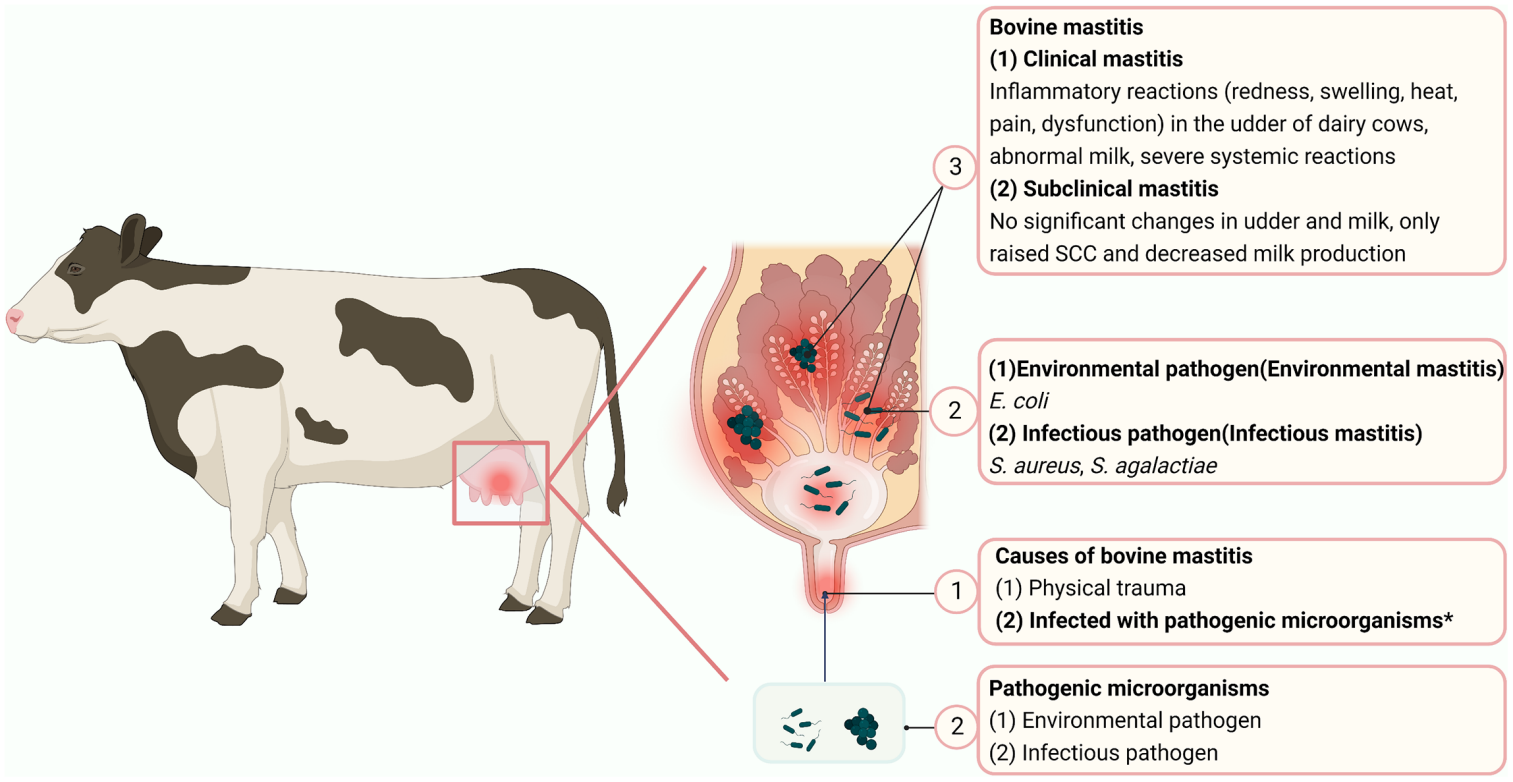
Mastitis in dairy cows. The circled numbers are to guide the reader through the sequence of mastitis. “*” represents the main causes of mastitis in cows (Created with BioRender.com).

Despite broad consumption of milk and milk products, mastitis in dairy cows, typically incited by bacteria (5), raises many concerns about milk quality. Mastitis can be divided into 3 stages: invasion, infection (colonization) and inflammation (6). Mastitis is classified as subclinical or clinical, based on whether clinical signs are absent of present. Subclinical mastitis causes some changes in the milk, including a white blood cell count > 500,000/mL (7), whereas cows with clinical mastitis may exhibit milk clots, udder swelling and systemic symptoms to varying degrees (8). Mastitis caused by infectious pathogens, including *Staphylococcus aureus*, *Streptococcus agalactiae* and *Mycoplasma bovis*, is infectious (9) (Figure 1), whereas mammary infections caused by environmental pathogens such as *E. coli*, *Klebsiella pneumoniae* and coagulase-negative *staphylococci* are called environmental mastitis (10, 11).

Current mastitis treatment relies on antibiotics and is the most important reason for antibiotic use in dairy cows. However, emergence of drug-resistant strains is threatening viability of antibiotics for mastitis treatment. Antimicrobial resistance (AMR) occurs when pathogens are able to overcome effects of antibiotics that were originally effective. It was reported that AMR was first detected in penicillin resistance of *Streptococcus pneumoniae*, and the isolation rate of drug-resistant strains increased by 11 percent over the following decade (12). Genes responsible for drug resistance can be transferred between bacteria of different taxonomic and ecological groups by mobile genetic elements such as phages, plasmids, naked DNA or transposons (13). Thus, resistant strains of animal origin and resistant strains of human origin may interact and transfer resistance.

With emergence of drug-resistant strains signals, it is clear that antibiotics will no longer be fully effective against mastitis. This is attributed to decades of antimicrobial use and misuse in human and veterinary medicine (13). Consequently, there is a global focus on finding alternatives to treat bacterial diseases. Finland substantially reduced macrolide use, resulting in nearly a 50% decrease in erythromycin resistance (14). This was proof of concept that reducing antibiotic use can reduce AMR.

**Figure 2:**
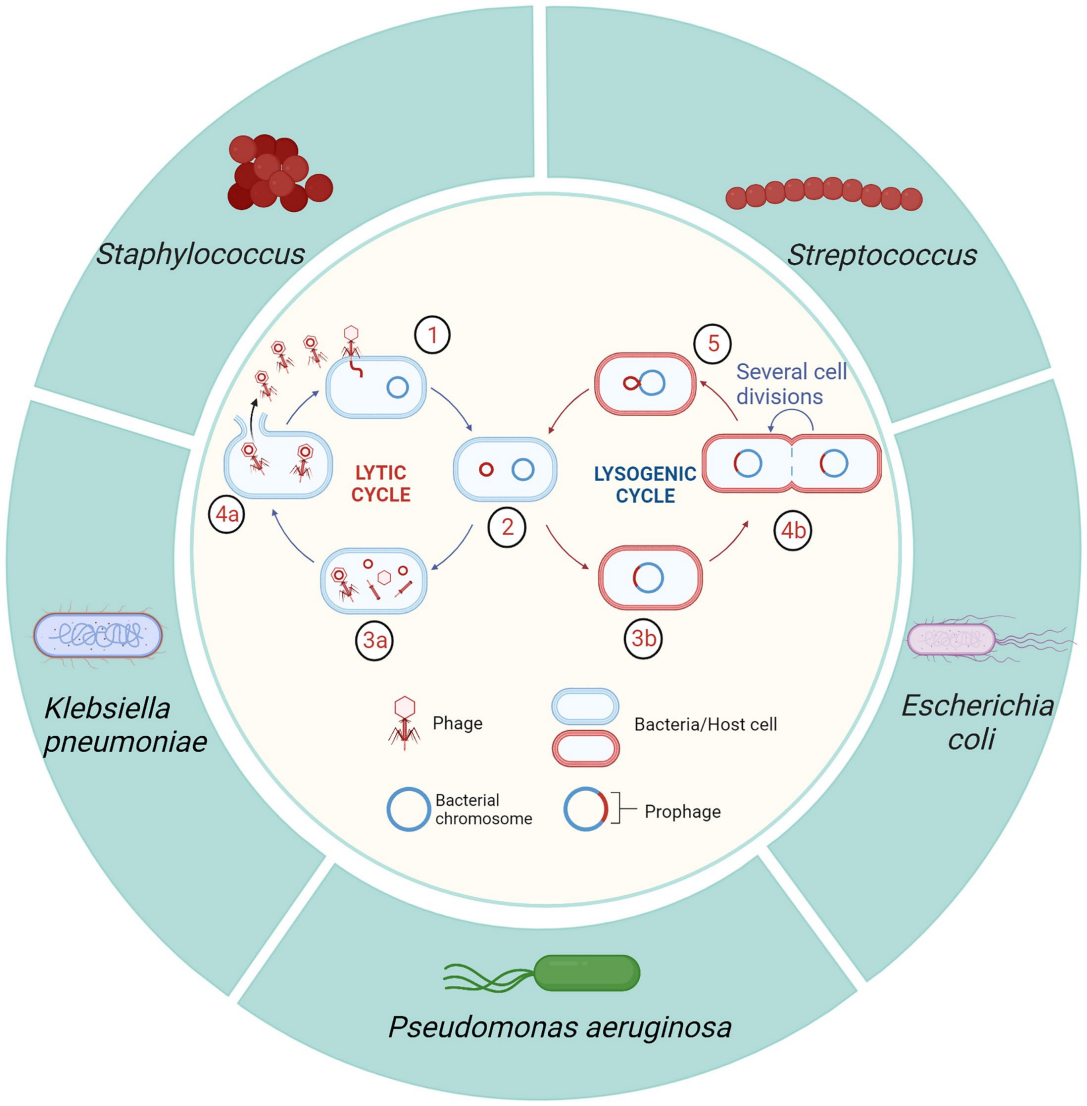
Annual antibiotic usage on dairy farms in the United States. Reprinted from de Campos et al. (15) under CC-BY-NC-ND.

In a study conducted on 40 large United States dairy farms, antibiotic treatment of dry cows and clinical mastitis cases accounted for > 75% of all antibiotic usage (15) (Figure 2). On dairy farms, direct costs of mastitis treatment include: drug treatment costs, veterinary service fees, and the value of discarded milk containing antibiotics or other ingredients (16). As antibiotics and veterinary fees account for 24% of financial losses from clinical mastitis (17–19), reducing antibiotic use could save considerable money. Combined with the presence of drug-resistant strains that prolong treatment, there is also a potential mortality and morbidity impact (13). In addition, as withdrawal of antibiotics for treating and preventing bovine mastitis presents substantial challenges for farmers, it is essential to provide viable alternatives.

Antimicrobial treatment of mastitis in dairy cows is generally regarded as necessary to maintain a balance among economics, animal welfare, and udder health (20). However, emergence of AMR strains is becoming one of the biggest threats to global health, food security, and societal development (21). Many mastitis-derived pathogens from various countries are resistant to common antibiotics (Table 1). Furthermore, common mastitis pathogens collected by our research team (Table 2) had a greater prevalence of AMR than mastitis pathogens from Europe (5, 36), confirming that mastitis caused by multi-drug resistant strains is a problem in large Chinese dairy herds (5).

**Table 1:** Antibiotic-resistant strains of bacteria causing mastitis in dairy cows.

Bacteria name	Antimicrobials																								Source	Reference
	P	AP	OX	AM	AC	CX	CR	CT	CE	CC	NE	G	KN	AI	CL	F	ER	TE	EN	CI	CO	VA	LN	M		
*Staphylococcus aureus*	100	100						100	100			33					44	83						67	Guanajuato, Mexico	(22)
	50				28			39	39			22	28		28		17	17		11		11		32	Canadian Bovine Mastitis Network	(23)
	32		100						100								56	56				72	60	NM	India	(24)
	90		75												40		NM	NM				NM	80	95	India	(25)
	93	87	47														70							100	Iran	(26)
	46	44	31									100			100		18	26		100				31	Malaysia	(27)
	86	86	100			100	98			98	90	100					90		98					NM	Rondonia, Brazil	(28)
*Streptococcus agalactiae*	5	5	5				100	100			16	3			100	1		21	100					1	Minas Gerais, Brazil	(29)
*Streptococcus dysgalactiae*	59				47			53	53	100					35		24	29		83		12		47	Canadian Bovine Mastitis Network	(23)
*Streptococcus uberis*	50				28			33	39	100					28		28	11		82		22		37		
	16		4				3	1									9	86	9					25	Lombardy, Italy	(30)
*Nocardia*	100	100	100	100	NM	100						100	100			100	100	100		100				100	Pernambuco, Brazil	(31)
*Escherichia coli*		90		95					33		26	37	32	37				90		5	NM			82	Bangladesh	(32)
		58		75	23			52				12	32		6			52	39		13			**93**	Algeria	(33)
		83										67	50							75				100	China	(34)
		76		NM				86				NM		NM	33			NM		NM				85	India	(25)
*Pseudomonas aeruginosa*									60			55		60						45	85			35	India	(35)
*Klebsiella pneumoniae*		93		79				NM				NM		NM	36			NM		36				50	India	(25)
CPS	33	22	89			89	89			89	77	89					89		89					NM	Rondonia, Brazil	(28)
CNS	69	69	96			96	96			92	89	100					96		96					NM		

(i) P Penicillin, AP Ampicillin, OX Oxacillin, AM Amoxicillin, AC Amoxicillin/Clavulanic Acid, CX Cefalexin, CR Ceftiofur, CT Cefotaxime, CE Ceftazidime, CC Clindamycin, NE Neomycin, G Gentamicin, KN Kanamycin, AI Amikacin, CL Chloramphenicol, F Florphenicol, ER Erythromycin, TE Tetracycline, EN Enrofloxaci, CI Ciprofloxacin, CO Colistin, VA Vancomycin, LN linezolid, M Multi-drug resistance, NM the results are not mentioned, CPS coagulase-positive Staphylococcus spp., CNS coagulase-negative *Staphylococcus* spp. (ii) Red color block-resistant, green color block-sensitive, blue color block-M, white color block-no detection. (iii) Numbers on red and green color blocks represent percentage of resistant strains and sensitive strains, respectively, and calculation of the drug resistance rate followed the principle of “rounding.”

**Table 2:** Comparison of drug resistance in common antimicrobial resistance (AMR) strains from bovine mastitis in China and Europe.

Pathogen	Antibiotic	Resistance rate (%)	
		China	Europe
*Staphylococcus aureus*	Penicillin	66	25
	Ceftiofur	16	1
	Oxacillin	18	2.6
	Tetracycline	17	5.2
CNS	Penicillin	62	29.1
	Enrofloxacin	23	5.5
	Oxacillin	84	56.4
	Tetracycline	34	7.3
*Escherichia coli*	Amoxi/CLA	81	3.9
	Ceftiofur	16	1
	Tetracycline	10	14.5
*Klebsiella* spp.	Amoxi/CLA	38	4.6
	Ceftiofur	21	0
	Tetracycline	32	19.5

CNS: coagulase-negative staphylococci; Amoxi/CLA: amoxicillin and clavulanic acid.

Given the urgency to reduce antibiotic use in the dairy industry, we review options for mastitis treatment and prevention, with goals of reducing emergence of antibiotic-resistant strains and minimizing financial losses. In this paper, we review clinical effects and application prospects of non-steroidal anti-inflammatory drugs (NSAIDS), herbal medicines, antimicrobial peptides (AMPs), bacteriophages (and phage endolysins), vaccination and other emerging therapies for treatment of bovine mastitis. Vaccination, herbal medicines, and AMPs can prevent mastitis by regulating the immune system. In addition, herbal medicines and AMPs can also act directly on bacteria to produce therapeutic effects. Phage (and phage endolysins) and NSAIDS are more effective on treatment. It is worth mentioning that although they can prevent and/or treat mastitis, the immature clinical application means that they are still an alternative to the prevention and treatment of mastitis.

## 2. Non-steroidal anti-inflammatory drugs therapy

Not all cases of clinical mastitis benefit from antibiotics, as 10–40% of cultures in clinical mastitis cases have no bacterial growth and do not require antibiotic therapy, and another 40% of positive cultures (mainly gram-negative bacteria and yeasts) are not sensitive to antibiotics approved for intramammary use (8). Intramammary antibiotic therapy is generally recommended only for infections caused by gram-positive bacteria such as *S. aureus*, *S. agalactiae* and environmental *Streptococci* spp. (37). In contrast, most Gram-negative infections are cleared by the cow’s own immune system (38). Therefore, antibiotics approved for use in the udder of dairy cows are effective in only 20–50% of clinical mastitis (8).

**Figure 3:**
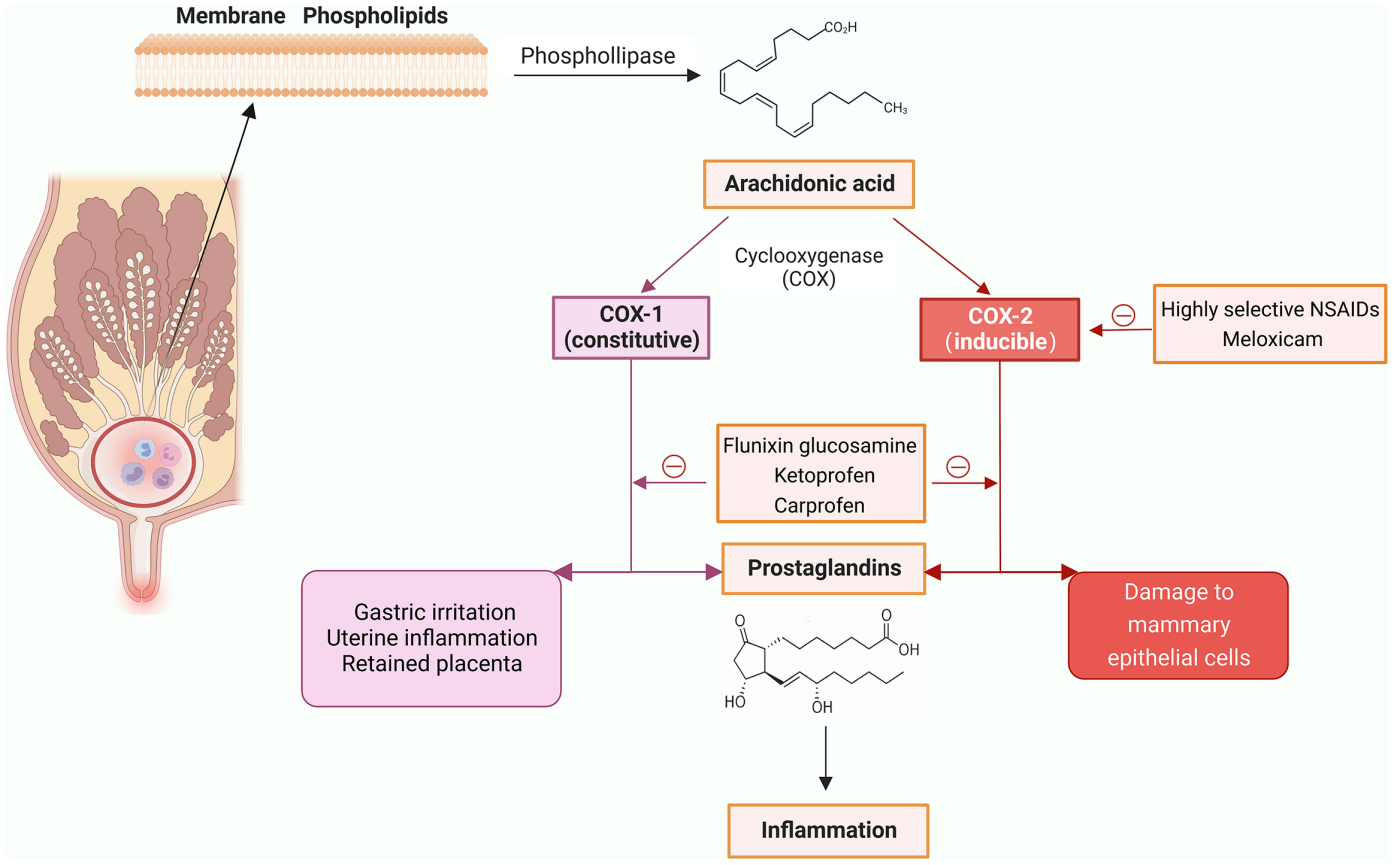
Mechanisms of action of non-steroidal anti-inflammatory drugs (NSAIDs) and commonly used NSAIDs (Created with BioRender.com).

The specific mechanism of action of NSAIDs is inhibition of cyclooxygenase (COX), reducing production of prostaglandins (an inflammatory mediator) (39). COX has 2 isoforms, COX-1 and COX-2; the former is naturally expressed in all tissues, and has a role in maintaining normal physiological functions, whereas the latter is induced by inflammatory stimuli and cytokines (40). NSAIDs that are more selective inhibitors of COX-2 have greater therapeutic effects, whereas those that are highly selective inhibitors of COX-1 have more side effects, including an increased risk of retained placenta, uterine inflammation, and gastric irritation (41) (Figure 3).

The NSAIDs used to treat bovine mastitis include flunixin meglumine, meloxicam, ketoprofen, and carprofen. Flunixin meglumine, the only NSAID approved by FDA in the US for dairy cows to control fever associated with mastitis and endotoxemia associated with *E. coli* mastitis, is commonly used as an analgesic in US food animals (42, 43). It inhibits both COX-1 and COX-2, but is more selective for COX-1, thereby increasing risk of retained placenta and digestive disorders (41). However, using only a single dose of flunixin meglumine can reduce these side effects (44). In cows with lipopolysaccharide-induced mastitis, flunixin meglumine increased feeding time and rumination during the first 9 and 12 h, and improved ruminal activity (45, 46). In addition, flunixin meglumine decreased blood nonesterified fatty acids and Isop concentrations in cows with *E. coli* mastitis, indicating a reduced inflammatory response (45).

Meloxicam is a more selective inhibitor of COX-2, greatly avoiding side effects associated with COX-1 inhibition (47). In a randomized trial on 2,653 cows from 20 herds, 1 mg/kg meloxicam orally at calving reduced the incidence of subclinical mastitis, increased feed intake and milk production, and reduced systemic inflammation (48). Furthermore, meloxicam alleviated the pain of LPS-induced clinical mastitis, mitigated udder edema, and reduced rectal temperature (49). When meloxicam was used to treat mild to moderate mastitis in the first 120 days of lactation, calving interval of infected cows were reduced, and the conception rate of infected cows was improved, which had positive benefits for pasture-based dairy production (50).

Ketoprofen inhibits both COX-1 and COX-2 (51) and has been used for treatment of bovine mastitis due to its rapid onset of action, short plasma half-life, low toxicity, and no milk withdrawal. It has been approved for use in Canada, Brazil and other countries (52). Intramammary administration of ketoprofen reduced SCC and damage to the blood-milk barrier, decreasing concentrations of IgG in milk during LPS-induced mastitis (53). Ketoprofen alone had positive effects on chronic mastitis (54), although effects on acute mastitis were less clear (52, 55).

Carprofen, like meloxicam, is a COX-2 selective, single-dose, long-acting NSAID to treat bovine mastitis (42, 56). In cows with mastitis, carprofen reduced heart rate, rectal temperature and udder swelling (57). In cows with *E. coli* mastitis, carprofen reduced rectal temperature and promoted ruminal motility (58).

There is a growing recognition of NSAIDs to manage inflammation, pain and endotoxin production in cows with mastitis (59). In Denmark, 72% of veterinarians use NSAIDs alone for mastitis, especially if caused by gram-negative bacteria (60). Some NSAIDs synergize with antibiotics in treatment of mastitis, such as meloxicam or ketoprofen plus gentamicin (59). In addition, some NSAIDs (e.g., meloxicam) can block virulence genes, prevent hemolysis, downregulate expression of genes related to biofilm formation, and inhibit *S. aureus* growth (59). We inferred that NSAIDs have potential to fully substitute for antibiotics in treating mastitis in cows in the absence of bacterial growth or for most gram-negative infections. Furthermore, since the primary mechanism of action for NSAIDs against bovine mastitis is non-bacterial, resistant strains should not affect efficacy.

## 3. Herbal medicines

Herbal medicines are derived from natural plants and have a long history of medicinal value, with limited or no side effects compared to antibiotics. The medicinal value of herbs are often due to their metabolites (e.g., phenolic acids, alkaloids, flavonoids, terpenoids, and volatile oils) that have antibacterial, antioxidant, and anti-inflammatory capabilities (61).

Many herbal medicines have antibacterial ability. For example, *Red ginger* had good bactericidal effects on *Staph epidermidis*, *S. aureus*, and *S. agalactiae* derived from bovine mastitis (62); the bactericidal mechanism is curcumin and gingerol that kill bacteria by disrupting their extracellular membrane (62). Biofilm is a key virulence factor to increase resistance of mastitis-derived methicillin-resistant *S. aureus* (MRSA); however, *Maize whiskers* significantly inhibited biofilm production by MRSA strains (63). Essential oils are secondary metabolites of plants with antimicrobial properties that do not stimulate drug resistance with prolonged use (64). Essential oils (*Oregano* essential, *Thyme* essential, *Carvacrol* essential, and *Thymol*) killed more than 30 species of *Staphylococci* (64). Several other herbal medicines and their extracts, including *Terminalia Chebula*, *Purslane* and *Dandelion* also had bactericidal activity against various mastitis pathogens (65).

Mastitis occurs when the immune system of the mammary gland fails to defend against bacterial invasion; therefore, it is very important to enhance immune activity to prevent and treat mastitis. *Dandelion* has free radical scavenging, antioxidant, antibacterial, and anti-inflammatory functions (66) and in a murine mammary gland infection model with *S. aureus, Dandelion* downregulated the inflammatory response (67). *Vitexin* treatment increased T-AOC, SOD, GSH-PX, CAT enzyme activity during *S. aureus* infection, both *in vitro* and *in vivo* (68). Baicalin, the bioactive component of *Scutellaria baicalensis georgi*, reduced expression of inflammatory factors and apoptosis of bMECs in cows with LPS-induced mastitis. Baicalein protected the mammary gland, reducing mastitis-induced damage (69, 70). The curative effect of mangostin on LPS-induced mastitis was attributed to suppression of inflammatory cytokine production, particularly the NF-κB and NLRP3 inflammasome (71). Geniposide anpolydatin was anti-inflammatory by interfering with expression of TLR4 and TLR2 and reducing expression of TNF-α, IL1β, and IL-6 (72, 73).

Immunity has a decisive role in occurrence, development and clearance of mastitis. Cows with robust immunity are often able to clear pathogenic bacteria during invasion of the udder. In addition to their powerful antibacterial influence, essential oils can be used as an alternative to antibiotics to improve feed efficiency, nutrient use, and animal health (64, 74). Dietary supplementation with black seed oil, chamomile oil, or cretian origanum oil starting 8 weeks before calving enhanced immunity in dairy cows (74). Furthermore, addition of essential oils to cow diets improved milk production, milk quality, udder health, and immunity (74). A Chinese herbal preparation containing 18 herbal medicines, including *Astmgali radix*, *Platycladi cacumen*, *Crataegi fructus*, and *Chuanxiong*, greatly promoted productivity in late-lactation cows exposed to heat stress (75).

In summary, herbal medicines contain bioactive components with great value in preventing and treating bovine mastitis, with mechanisms of action similar to antibiotics, but without the presence of antibiotic residues in milk (76). However, some bacteria are naturally resistant to herbal compounds and others develop resistance over time (77–79). Moreover, few herbal medicines have been approved by the FDA for clinical use, mainly due to the complexity of their composition and the difficulty to accurately assess efficacy and safety (80), although at least some of these issues can be readily addressed.

## 4. Antimicrobial peptides therapy

Antimicrobial peptides are another promising replacement for antibiotics. Most cells produce naturally occurring antibiotic-like molecules, known as AMPs, key components of innate immunity (81). Their antimicrobial activity is attributed to net charge, hydrophobicity, and amphiphilicity (82). As of December 2022, the continuously updated Antimicrobial Peptide Database (APD, https://aps.unmc.edu/home) included 3,425 AMPs from 6 kingdoms, 147 human host defense peptides, 385 bacteriocins/peptide antibiotics isolated/predicted from bacteria, 5 from archaea, 8 from protozoa, 25 from fungi, 368 from plants, and 2,489 from animals, including some synthetic peptides.

Nisin, a natural antimicrobial peptide produced by *Lactococcus lactis*, had excellent antimicrobial activity against gram-positive bacteria isolated from mastitis in dairy cows (83). In a bovine mastitis trial, there was no difference between Nisin and an antibiotic group for rates of bacteriological or clinical cure (84). An isolate of *S. aureus* from mastitis that was resistant to a variety of antibiotics was readily killed by Nisin (84). For treatment of subclinical mastitis, Nisin not only reduced somatic cell count, but also had good bacteriological cure rates against *S. agalactiae*, *S. aureus*, and coagulase-negative *Staphylococci* (CNS) (85).

Polybia MP-1, a 14-amino acid AMP from wasp venom, was bactericidal against multidrug-resistant *S. aureus*, *E. coli* and *K. pneumoniae* strains from bovine mastitis (25, 35). Esculentin 1–21, an AMP from frog skin, had broad-spectrum antimicrobial activity (86), particularly against *Pseudomonas aeruginosa*, *E. coli*, and *S. agalactiae in vivo* and *in vitro* (87). In a clinical trial, Esculentin 1–21 had a 100% improvement rate after 5 days without side effects (87). Although an increasing number of mastitis-derived strains have multidrug resistance, AMPs had good bactericidal ability against them.

AMPs secreted in the mammary tissue of cows include β-defensins, psoriasin, cathelicidins, and lactoferrin (88). Bactericidal and therapeutic functions of AMPs secreted by mammary gland of cows, especially β-defensins (89), have been studied. Tracheal antimicrobial peptide (TAP), a cationic β-defensin, can be produced by bMECs (90). In both *in vitro* and *in vivo* infection models, TAP effectively killed *S. aureus* and reduced induced apoptosis of bMECs (91). Plectasin, a cationic AMP with 40 amino acids isolated from fungus, has low cytotoxicity (92). MP1102 is similar to plectasin and had strong antibacterial activity against MRSA, even inside bMECs (66, 93). Recently, a series of specific and targeted antimicrobial peptides based on the pheromone and cell-penetrating peptides of *S. agalactiae* were produced and designated cell-penetrating selective antimicrobial peptides L1–L12 (94). L1, L2, and L11 killed *S. agalactiae* by membrane disruption, whereas L2 and L10 entered cells and activated endocytosis (94).

Although AMPs secreted directly from mammary tissue have tissue homology and high bactericidal efficiency, purification methods, production, and *in vitro* preservation stability restrict clinical applications (95). Therefore, future research should use eukaryotic expression vectors or genetic engineering to develop novel AMPs based on natural AMPs. Bacteria can become resistant to AMPs, and potential cross-resistance between AMPs and conventional antibiotics has been reported (23). Gram-negative bacteria can resist effects of AMPs by surface remodeling, biofilm structure, efflux pumps, interception (binding and isolation of antimicrobial peptides so that they cannot act on the bacterial membrane), proteolytic degradation, and modulation of cationic antimicrobial peptides expression (96).

## 5. Bacteriophages

**Figure 4:**
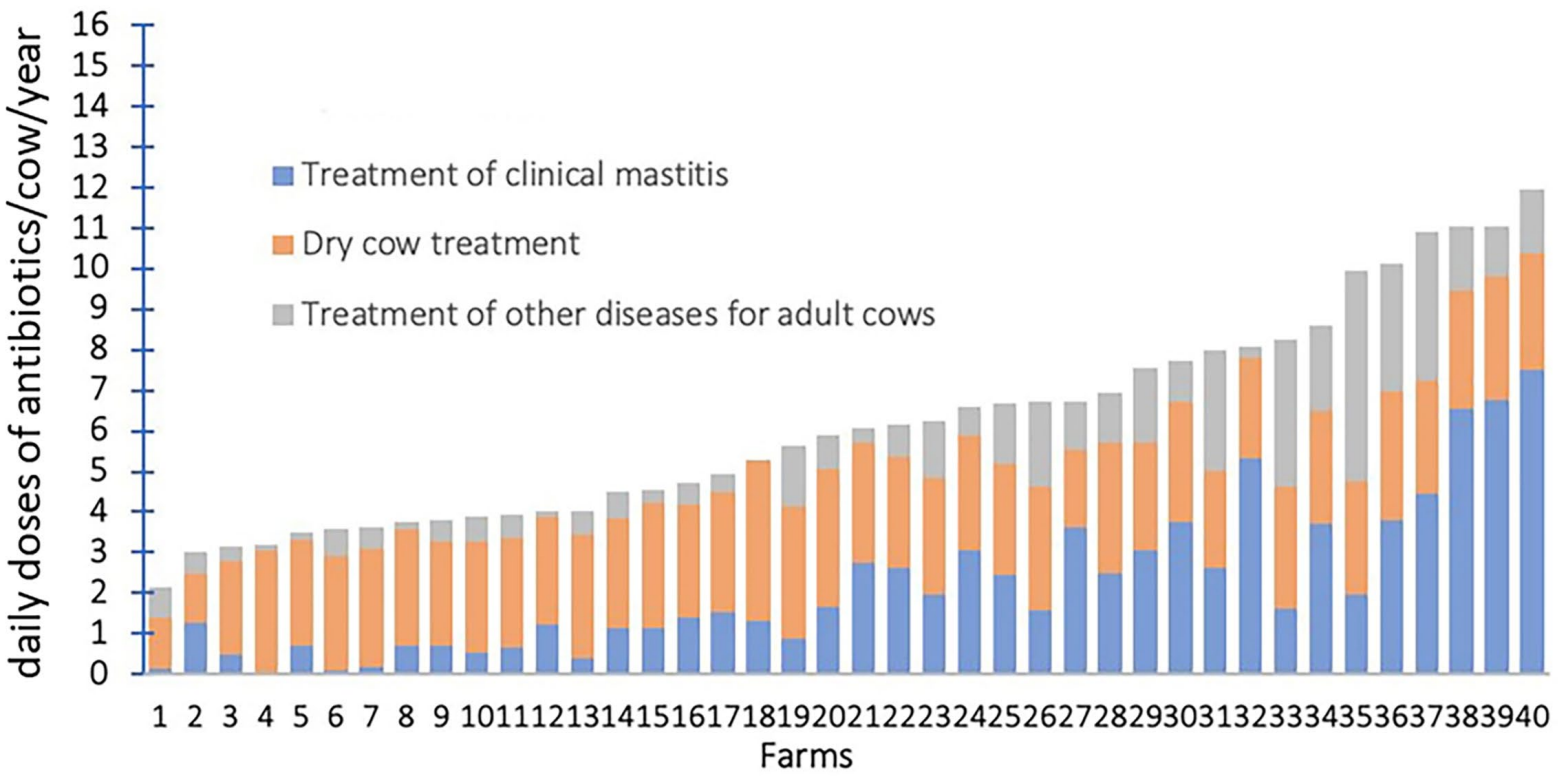
Mechanism of phage lysis of host bacteria and published types of mastitis pathogenic bacteria targeted by phages. 1. Phage attaches to host bacterium and injects DNA. 2. Phage DNA enters the lytic or lysogenic cycle. 3a. DNA and protein synthesis followed by assembly of new phages. 4a. Lysis of the host bacterium, releasing a large number of new phages. 3b. Phage DNA is integrated into the host bacterium chromosome. 4b. Lysogenic bacterial have normal reproduction. 5. Under specific conditions, the prophage is isolated from the host bacterium genome and enters the lysis cycle (Created with BioRender.com).

Bacteriophages are viruses that can lyse bacteria; based on their life cycle, they are classified as either lytic or lysogenic (temperature) phages (97). Lytic phages usually attach an adsorption structure to a specific receptor on the surface of the bacterium, inject DNA into the host bacterium through the tail structure, lyse the host, and release a large number of phages (98). Unlike lytic phages, lysogenic phages assemble their own genes in the host bacterial genome and coexist without causing host bacterial lysis (98) (Figure 4). Due to their direct bactericidal effect, lytic phages are preferred for treatment of bacterial infectious diseases. In contrast, lysogenic phages are usually used as vectors to transmit genes encoding inhibition of bacterial virulence, to develop small molecular proteins of simulated bacteriophage derivatives for bacterial virulence, and to design vaccines (99). Phages are usually safe, effective, non-residual, highly specific, and without effect on non-target bacteria, with great potential to replace antibiotic therapy for mastitis in cows. Two strains of *S. aureus* phages, SAJK-IND and MSP, were isolated from mastitis milk and environmental sewage and were 100% bactericidal against 120 *S. aureus* strains (100). In another study, 36 MRSA strains were isolated from milk and teat skin of cows with subclinical mastitis and had 100% susceptibility to *S. aureus* phages (22). Teng et al. (101) isolated *S. aureus* phage 4086-1 from mastitis milk, which efficiently killed MRSA in the murine mammary gland and had a good therapeutic effect. *S. aureus* phages ΦSA012 and ΦSA039 had broad lytic spectrums (102). *In vivo*, phage ΦSA012 removed *S. aureus* from the murine mammary gland, suppressing the inflammatory response and tissue damage (102).

Regarding the use of phages to control other pathogens causing bovine mastitis, Bai et al. (103) isolated a *S. agalactiae* phage JX01, reported its complete genomic sequence, and determined it can lyse 65.3% of bovine *S. agalactiae* with no killing effect on human or fish strains (i.e., high specificity). The T4 phage vB_EcoM-UFV13, a novel *E. coli* phage with a broad host range, decreased the bacterial load by 90% in murine mammary glands and had a positive result on *E. coli*-induced mastitis in dairy cows (104). PAJD-1, a phage isolated from sewage samples on a dairy farm, lysed 80% of *P. aeruginosa* strains (105). The edema and hemorrhagic response of mouse mammary tissues caused by *P. aeruginosa* was greatly alleviated by the action of PAJD-1 *in vivo*, similar to antibiotics (105). Our research team isolated 5 strains of *K. pneumoniae* phages from the sewage samples of dairy farms, and conducted biological identification, genome sequencing and therapeutic research (106–108). We reported that *K. pneumoniae* phages mitigated *K. pneumoniae*-induced inflammation in bMECs and reduced structural damage and inflammatory responses of murine mammary gland tissue (107, 108).

The law of survival of the fittest suggests that the coexistence of phages and bacteria for millions of years results from their co-evolution, i.e., the phage cannot completely eliminate the host bacterium because there is always a portion of the host bacterium that has evolved into a mutant strain that is unaffected by the phage (109). Notwithstanding the superior lytic competence of phages on pathogenic bacteria, even strains in the biofilm state, there is no shortage of phage mutants (110). In that regard, it was stated that *E. coli* can develop resistance to phages within a short interval (111). Experiments by Pires et al. (112) also noted development of resistance. Furthermore, after 24 h of phage action, *P. aeruginosa* developed two strains of bacteriophage-insensitive mutants (BIM). The co-evolutionary nature of phages and bacteria coupled with the abundance and diversity of phages in nature may be a critical solution to addressing bacterial resistance to phages (113). One method is to replace the phage to which the bacteria have developed resistance, and another approach is to use a cocktail of multiple phages with different receptors and complementary hosts (110). Phage cocktails can not only expand the scope of response but diminish emergence of phage-mutant strains (114). Phage cocktails are mixtures of phages that broaden the host range and minimize production of phage-resistant bacteria (114). By mixing 3 strains of phages, Garcia et al. (115) demonstrated the bactericidal power of the cocktail was significantly enhanced. In treatment of mastitis, an *E. coli* cocktail consisting of phages (SYGD1, SYGE1 and SYGMH1) had more powerful bactericidal activity and clinical therapeutic effect than a single phage (34).

Endolysins encoded by phages also have strong potential for clinical application due to broad lyase spectrum, safety and stability (116). PlySs2 and PlySs9, 2 bacteriocins from *Streptococcus suis* prophage, had broad lytic activity against *Streptococcus uberis* isolated from bovine mastitis (117). LysRODI, encoded by the *Staphylococcal* phage phiIPLA-RODI, had superior lysis capacity against *Staphylococci* strains from dairy farms and decreased mammary tissue damage caused by *Staphylococcus* infection in mice (118).

In recent years, more and more animal models and clinical trials have been conducted to evaluate the therapeutic effects of phages, and some phage products have been approved for clinical treatment. However, there are still many difficulties in using phages as first-line agents, due to: (i) lack of chemotaxis, preventing phages from dispersing and reaching sites of infection (102); (ii) intravenous administration of phages is limited by the body’s immune system and focused on direct action at the site of infection, with deep tissues and intracellular bacteria being less accessible (119); (iii) phages are replication-competent nucleoprotein complexes, and their “pharmacology,” e.g., dose, is not well understood (120); (iv) the safety of phage products is affected by many elements, e.g., purity and sterility (121); (v) phages have not yet reached a gold standard for double-blind efficacy assays (122); and (vi) phage therapies do not yet have a dedicated legal regulatory framework and have only been implemented in a few countries (121, 123).

## 6. Vaccination

Effective vaccines can reduce the incidence of mastitis, thereby effectively reducing antibiotic use. Vaccines have been developed for some pathogens causing clinical mastitis, e.g., *E. coli*, *S. aureus*, and *Streptococcus* spp. Among them, J5 mutant strains-based vaccines represent a breakthrough in *E. coli* vaccine development (124). In clinical trials, *E. coli* J5 vaccination reduced the incidence of gram-negative mastitis in dairy cows, with protection lasting up to the third month of lactation (125). In another study, J5 vaccination failed to reduce the incidence of *E. coli* mastitis, although it mitigated severity (126). Vaccines for controlling *S. aureus* mastitis consist of either whole cells (autologous vaccines) or subunits (recombinant proteins and bacterial surface extracts) (124). Small colony variants of *S. aureus* have potential for development of a live vaccine capable of preventing mastitis in dairy cows. Côté-Gravelet et al. (127) developed a novel attenuated mutant by knocking out the *hemB* and *vraG* genes and demonstrating its potential as an attenuated vaccine for ameliorating udder infections caused by *S. aureus*. An experimental vaccine based on *S. aureus* surface-associated protein had promise, enhancing serum-associated protein titers and maintaining efficacy for ~ 4 months (128). Another study used recombinant protein technology to confirm that genes associated with iron acquisition had good immunogenicity in both rabbits and cattle. 54 strains of *S. aureus* were screened for 5 iron acquisition system-related genes: *isd*, *feo*, *sir*, *sst* and *fhu*. IsdH protein from the *Isd* system induced a long-lasting immune response when inoculated in cattle, implying IsdH was a good candidate for a *S. aureus* mastitis vaccine (129). *Streptococci* species closely associated with mastitis in dairy cows are primarily *S. uberis*, and also *S. agalactiae* and *Streptococcus dysgalactiae* (130, 131). By using the strain of *S. uberis* that formed the greatest biofilm as the source of the vaccine, Collado et al. (132) evaluated a subunit vaccination based on lipophosphatidic acid (LTA) for *S. uberis* against experimental intra-dairy heterozygous strains of infection in dairy cows. Protection was incomplete, but vaccination significantly reduced clinical signs and hastened recovery of the milk compared to the control group (132). Cows given live *S. uberis via* subcutaneous injection had higher serum antibody titers and less severe clinical signs compared to unvaccinated cows (133). However, this vaccine was effective against homologous but not heterologous strains (133).

Vaccines have much potential for preventing mastitis in dairy cows. However, it is evident that the number of pathogenic bacteria causing mastitis in cows far exceeds bacteria targeted by existing vaccine development. Furthermore, pathways and mechanisms of infection for these pathogenic bacteria are not uniform, posing challenges to developing effective vaccines for mastitis in cows. Additionally, there are numerous constraints, such as timing of administration and duration of effect.

## 7. Other therapies

### 7.1. Probiotics

That intestinal flora can induce bovine mastitis through endogenous paths highlights potential to use probiotics to treat mastitis in dairy cows (134). Feeding *Bacillus subtilis* to heifers and transitional cows *subtilis* for 3 weeks before calving and throughout lactation reduced the incidence of clinical mastitis, SCC, and days of discarded milk (135). Furthermore, *Lactobacilli*, Yeast, and LAB (a mixture of *Lactobacilli* and maltodextrin) optimized the mammary microbiota and increased mammary resistance of dairy cows (136). *Lactobacillus casei*, a probiotic that regulates the digestive system, can adhere to and internalize into bMECs without altering cell viability and morphology, but prohibiting *S. aureus* infection (137). Moreover, *L. casei* activated innate immunity of bMECs and reduced susceptibility to infection (138). A commercial post-dip solution containing *L. casei*, *L. brucei*, and *L. paracasei* has been used on dairy farms and reduced the incidence of mastitis (139).

### 7.2. Stem cells

Mesenchymal stem cells (MSCs) are non-specialized pluripotent cells capable of self-renewal and differentiation into specific cell types, with potential for tissue regeneration. As they are easily accessible, their therapeutic competence is of interest (140). MSCs from fetal bovine bone marrow (BM-MSC) and adipose tissue (AT-MSC) reduced growth of *S. aureus in vitro* (141). Intramammary administration of AT-MSC in dairy cows killed *S. aureus* in the udder without side effects (142). A recent study used MSCs from umbilical cords and their extracellular vesicles to treat subclinical mastitis (143). MSCs may have an immunomodulatory role by releasing bioactive components and promoting repair of damaged tissues in dairy cows with mastitis (142, 143).

### 7.3. Nanotechnology-based therapy

Nanotechnology-based drug delivery enables drugs to be deposited, sustained and slowly released at target locations, thereby overcoming some limitations of conventional drugs, including antibiotic resistance (144). Self-assembly tilmicosin nanogel was used on cows with *S. aureus* mastitis and had a higher cure rate compared to a conventional treatment group (145). Cinnamon oil and silver nanoparticles were bactericidal against *S. agalactiae* (146). Polyherbal nanocolloids from *Dandelion*, *Cinnamon*, *Phyllanthus emblica*, *Terminalia*, and *Citronella* had efficient, dose-dependent antibacterial ability against mastitis-derived pathogens (147).

### 7.4. Photodynamic therapy

Photodynamic therapy (PDT) has much potential for treating bovine mastitis (148). A non-toxic photosensitizer is activated to produce ROS that kills bacteria by altering its cell membranes and DNA (149). In cows with subclinical mastitis, PDT was bactericidal against *S. uberis* and coagulase negative *S. aureus* (CNS) (150). Furthermore, in sheep with mastitis, PDT reduced CNS, *Streptococcus* spp. And *E. coli* within udders (151). Though PDT has much promise to treat mastitis, the method is still in initial research stages. Improvements in the photosensitizer, light sources and oxygen supply are needed to strengthen the effectiveness of action and reduce adverse side effects (152).

### 7.5. Acoustic pulse therapy

Acoustic pulse therapy (APT) is another antibiotic-free strategy to treat bovine mastitis. Cows with mastitis can be treated by APT devices using low-power acoustic pulses to penetrate deep tissue and disperse pressure waves over a broad region of udders (153). In addition, APT can activate immune cells and repair damaged tissue (153). Similarly, APT was more effective for treating mastitis caused by *E. coli* compared to *Streptococcus* (154), with APT-treated cows producing an addition 500 L milk in a 305-day lactation (154).

## 8. Conclusion and future prospects

Commercial dairy farms are likely to have ongoing issues with mastitis. At present, antibiotic therapy is the first line of treatment, but there is much concern about emergence of multi-drug resistant strains on dairy farms and the potential for that resistance to be spread to pathogens affecting humans. Therefore, there is a great impetus to identify alternatives for treating mastitis in dairy cows.

From our perspective, NSAIDs, herbal medicines, AMPs, bacteriophages and vaccination have much potential for easing the plight of antibiotic resistance. Many veterinarians already use NSAIDs as adjunctive therapy for mastitis. Furthermore, some practitioners are using NSAIDs alone for treating mastitis. The advantages of herbal medicines are undeniable, but much effort is needed to produce commercially viable products. Although AMPs can also have positive effects, their ability to damage eukaryotic cells needs to be addressed. Phages are well known for their selective effects on target bacteria, making them the most prospective successor to antibiotics for bovine mastitis. In fact, they have already contributed to save many lives in human infection. Vaccines can prevent mastitis, limit the severity of clinical signs, and hasten cure. However, due to the wide range of mastitis-causing organisms, especially environmental pathogens that are becoming dominant, vaccine control of mastitis faces many challenges. Some other possibilities were also briefly described in the review. Probiotics may work by modulating the intestinal flora, with a proof of concept of the direct effect of probiotics on mastitis in dairy cows. Although at the initial stage of research, nanotechnology has great application potential in the treatment of mastitis in combination with other antibacterial substances due to their good drug-carrying capacity. PDT and APT are emerging as potential approaches in the treatment of mastitis in dairy cows, but more research is needed to make them practical and effective.

## Author contributions

XL wrote the manuscript with support from CX, BL, JK, BH, XT, and JG. All authors contributed to the article and approved the submitted version.

## Funding

This study was financially supported by the Beijing-Tianjin-Hebei Collaborative Innovation Community Project (21346601D) and the National Natural Science Foundation of China (32273082 and U21A20262).

## Acknowledgments

We thank Professor Yuxiang Shi from Hebei University of Engineering for providing the current status of antibiotic use on many dairy farms.

## Conflict of interest

The authors declare that the research was conducted in the absence of any commercial or financial relationships that could be construed as a potential conflict of interest.

## Publisher’s note

All claims expressed in this article are solely those of the authors and do not necessarily represent those of their affiliated organizations, or those of the publisher, the editors and the reviewers. Any product that may be evaluated in this article, or claim that may be made by its manufacturer, is not guaranteed or endorsed by the publisher.

## Abbreviations

AMP: antimicrobial peptides
AMR: antimicrobial resistance
APT: acoustic pulse therapy
BIM: bacteriophage-insensitive mutants
bMEC: bovine mammary epithelial cell
CNS: coagulase-negative *Staphylococci*
COX: cyclooxygenase
MRSA: methicillin-resistant *S. aureus*
MSC, BM-MSC, AT-MSC: mesenchymal stem cells, or fetal bovine bone marrow, or adipose tissue
NSAID: non-steroidal anti-inflammatory drug
PDT: photodynamic therapy
SCC: somatic cell count
